# Influence of Chagas Disease on the Pharmacokinetics of Benznidazole in the Dog Model

**DOI:** 10.1021/acsptsci.5c00571

**Published:** 2026-02-18

**Authors:** Lorena Cera Bandeira, Leonardo Pinto, Fernanda de Lima Moreira, Glauco Henrique Balthazar Nardotto, Luciana da Fonseca Medeiros, Kátia Fonseca, Paula Melo de Abreu Vieira, Cláudia Martins Carneiro

**Affiliations:** † Laboratory of Immunopathology, Nucleus of Biological Sciences Research, 28115 Federal University of Ouro Preto, Ouro Preto 35400-000, Minas Gerais, Brazil; ‡ Laboratory of Pharmacometrics, Faculty of Pharmacy, 28125 Federal University of Rio de Janeiro, Rio de Janeiro 21941-599, Brazil; § Bioanalytics, Metabolomics & Pharmacokinetics Resources (BMPK), 2074 Roswell Park Comprehensive Cancer Center, Buffalo, New York 14263, United States; ∥ Department of Clinical Analysis, School of Pharmacy, Federal University of Ouro Preto, Ouro Preto 35400-000, Minas Gerais, Brazil

**Keywords:** Chagas disease, benznidazole, pharmacokinetics, cytokine, membrane transporters, dog model

## Abstract

The high variability in efficacy and safety of antichagasic chemotherapy involving benznidazole (BNZ) may be due to pharmacokinetic-related factors. The study evaluated the impact of experimental acute and chronic infections by the Berenice-78 strain of *Trypanosoma cruzi* on the pharmacokinetics of BNZ in dogs. Twenty-seven mongrel dogs were divided into the following groups: acute infection state treated with BNZ, chronic infection state treated with BNZ, acute and chronic positive controls (not treated with BNZ), and a healthy group treated with BNZ. They were evaluated at (1) basal state, (2) during infection without treatment, for cytokines panel (IL-6, interferon γ (IFN-γ), IL-10 and tumor necrosis factor α (TNF-α)) evaluation, and (3) during BNZ steady-state levels at 10, 30, 40, and 60 days after start of treatment with oral BNZ 3.5 mg/kg b.i.d. administration, in acute and chronic *T. cruzi*-infection, for BNZ pharmacokinetics and cytokines panel evaluation, and (4) 30 days after BNZ treatment end for cytokines evaluation. BNZ levels in serum samples were quantified using a curve range of 0.1–100 μg/mL in plasma by high-performance liquid chromatography with diode array detection (HPLC-DAD) analysis. Pharmacokinetic parameters remained stable during treatment phases, indicating no autoinduction or inhibition. Acute infection pharmacokinetics resembled that of healthy controls. Chronic infection significantly increased *C*_max_, *C*_ss_, and AUC_0–12_, while decreasing Vd/F and CL/F compared to both healthy and acute groups. Notably, IL-6 levels were elevated ∼7-fold during chronic infection. These data suggest that chronic inflammatory status modulates BNZ disposition, likely via IL-6 mediated P-glycoprotein inhibition. Understanding these pharmacokinetic changes is critical for optimizing BNZ dosing strategies in Chagas disease management.

## Graphical abstract

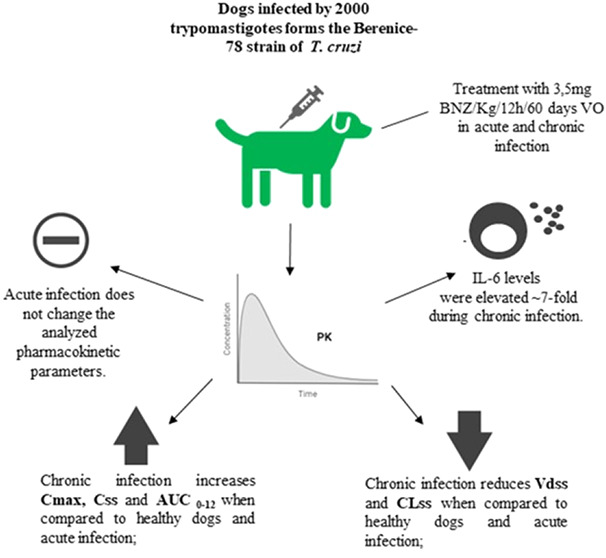

Chagas disease (ChD), also known as “silent and silenced disease”,^1^ is a tropical zoonotic infection caused by the parasite *Trypanosoma cruzi*. This disease has a highly complex pathogenesis and progresses through two distinct phases: the acute phase and the chronic phase. These phases vary significantly in their immunopathological characteristics, inflammatory responses, and therapeutic effectiveness.^2^

It is estimated that the number of deaths related to ChD annually reaches 12,000 and that 75 million people are at risk of contracting the disease.^3^

Benznidazole (BNZ) is the first-line treatment for ChD in most countries.^4^ It is still not considered the ideal compound to achieve a cure for ChD due to many limitations that include variable therapeutic response with a parasitological cure in up to 80% of patients in the acute phase, but only 20% in the chronic phase of the disease,^5^ besides the resistance of the various strains and clones of the parasite and high treatment toxicity compromise the treatment with BNZ. Such treatment limitations may be related to its pharmacokinetic (PK) properties, such as low solubility and intestinal permeability, as a probable consequence of its physicochemical properties, the autoinduction of its enzymatic metabolization systems (hepatic CYP3A and intestinal GST) and/or transport (P-gP and MRP2).^6–10^ For the patient, these characteristics translate into low efficacy and high toxicity BNZ treatment with low adherence by the patient.

In this sense, preclinical pharmacokinetic studies are of great importance as they evaluate several parameters to identify possible causes of therapeutic ineffectiveness and toxicity. Scientific evidence indicates that the canine model is the most suitable experimental model for investigating the preclinical pharmacokinetics of BNZ, since there is similarity in the immunopathogenic mechanisms as well as the efficacy results of BNZ when compared to human ChD and therefore presents an important translational value.^2,11–15^

The present study is proposed to understand whether the inflammatory process by *T. cruzi* during acute and chronic infection is capable of influencing the kinetic disposition of BNZ in the dog model to contribute to the design of a more rational pharmacotherapy for patients with ChD.

## Results and Discussion

In this study, it was proposed for the first time to evaluate the influence of acute and chronic ChD infection on the pharmacokinetic parameters of BNZ in a dog model. For ChD, it is already well established by our research group that the dog model reflects pathophysiological aspects of *T. cruzi* infection and pharmacodynamic aspects of treatment with BNZ, similar to that observed in humans,^2,5,11^ therefore, a fundamental model in preclinical studies. So, in the present study, we evaluated the dog model as a suitable preclinical pharmacokinetic model in ChD under BNZ treatment in order to set this approach as a translational model in drug development in ChD. The infectivity and mortality rates in the investigated dogs were, respectively, 100 and 18% in the complete study of the chronic phase (*n* = 3, 1 Male, 2 Females), and the death of the animals occurred 2, 4, and 7 months after infection. Regarding the acute phase of the disease, the infectivity rate was 100%, and the mortality rate was zero. The dogs were divided into their respective experimental groups according to the infection state: ACUTE-BNZ, CHRONIC-BNZ, ACUTE-POS, and BNZ-POS (Figure 1).

**Figure 1:**
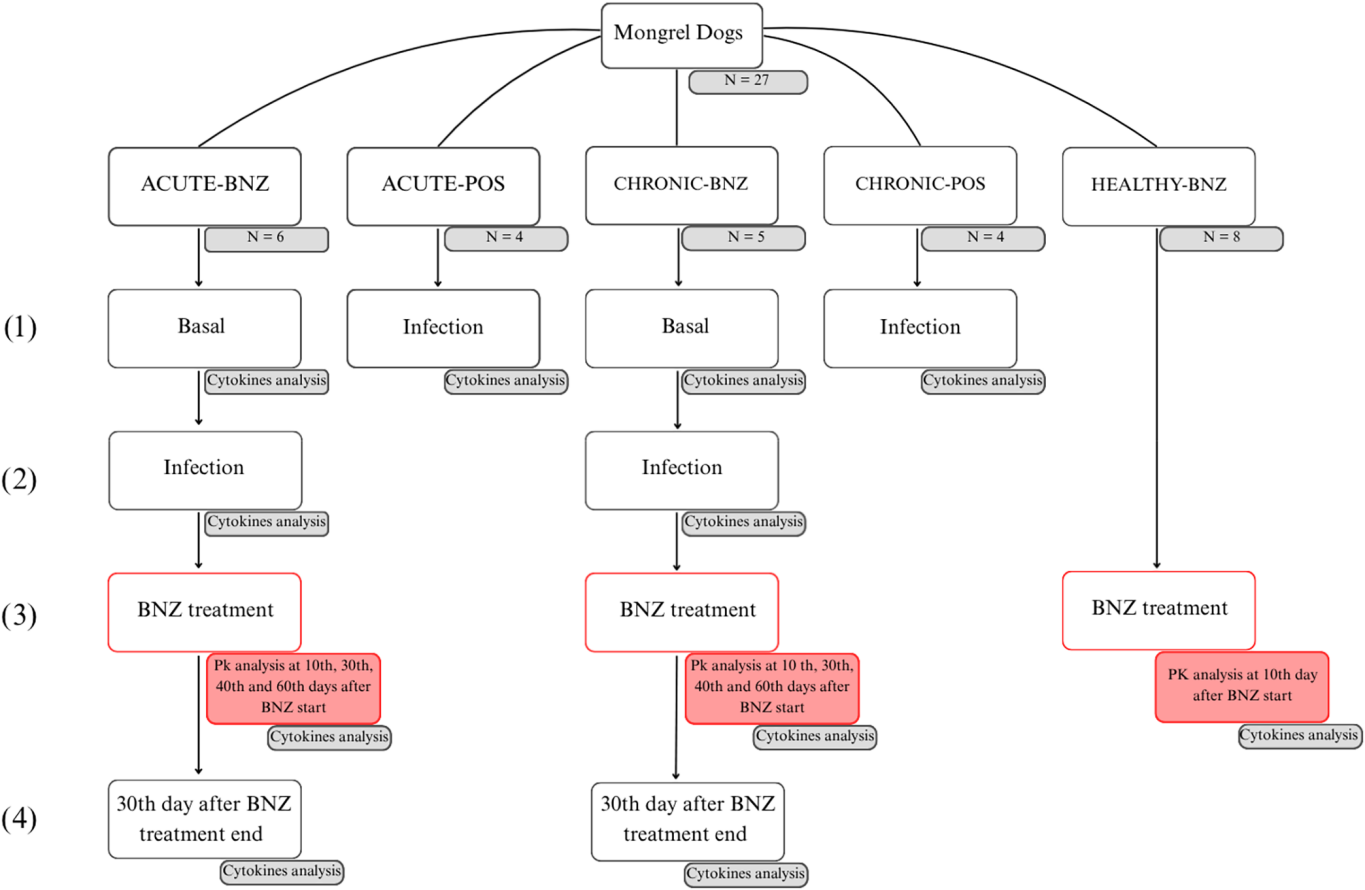
Study design. The experimental groups were acute infection state treated with BNZ (ACUTE-BNZ) (*n* = 6), chronic infection state treated with BNZ (CHRONIC-BNZ) (*n* = 5), acute (ACUTE-POS) (*n* = 4), and chronic (CHRONIC-POS) (*n* = 4) positive controls (not treated with BNZ), and a healthy group treated with BNZ (HEALTHY-BNZ) (*n* = 8). They were evaluated at (1) basal state for cytokines panel evaluation (IL-6, interferon γ (IFN-γ), IL-10, and tumor necrosis factor α (TNF-α)); (2) during infection without treatment for cytokines; (3) during BNZ steady-state levels at 10, 30, 40, and 60 days after start of treatment with oral BNZ 3.5 mg/kg b.i.d. administration for cytokines and/or BNZ pharmacokinetics evaluation; (4) 30 days after BNZ treatment end for cytokines evaluation.

All the dogs investigated were clinically evaluated before, during, and after each occasion of the acute and chronic phases and showed hematologic and biochemical values within normal limits for renal, hepatic, cardiac, and muscular functions. The body weight of the animals was also monitored throughout the treatment, with no significant differences observed between ACUTE-BNZ versus ACUTE-POS or CHRONIC-BNZ versus CHRONIC-POS groups. These data demonstrate that all dogs were maintained to be stable during the entire experimental protocol.

Figure 2 demonstrates the serum BNZ steady-state concentrations versus time profiles after oral 3.5 mg/kg b.i.d. administration in dogs evaluated at 10th, 30th, 40th, and 60th days after beginning of treatment in dogs under acute (ACUTE-BNZ group) and chronic (CHRONIC-BNZ group) infection and compared with healthy dogs (HEALTHY-BNZ).

**Figure 2:**
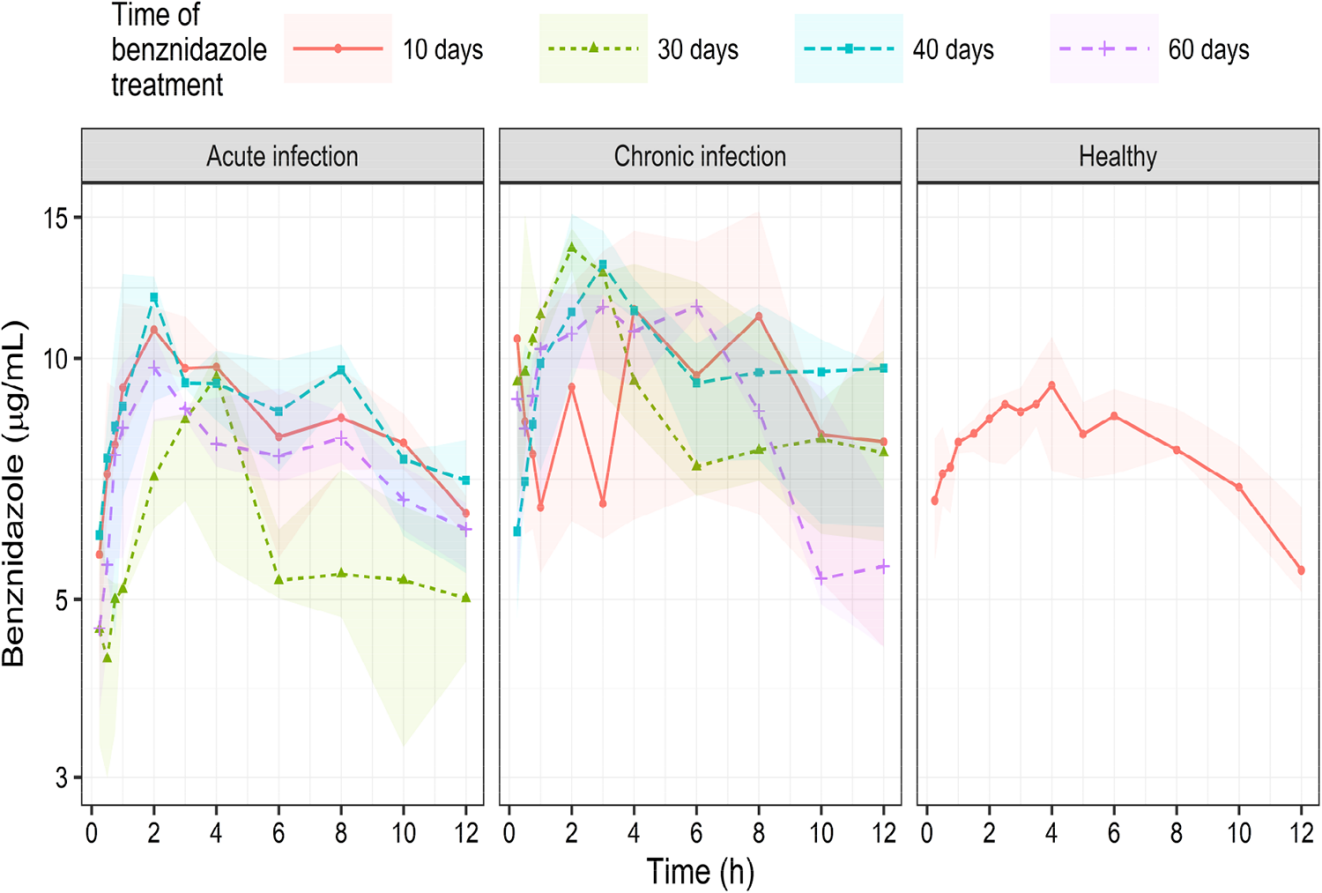
Benznidazole serum concentrations from 0 to 12 h, presented as medians (dots and lines) and 25–75 percentiles intervals (shaded area), in acute (*n* = 6) and chronic (*n* = 5) experimental infection with *T. cruzi* Be-78 strain infected following treatment with 3.5 mg oral benznidazole/kg b.i.d. administration for 10, 30, 40, and 60 days during steady-state levels and healthy dogs (*n* = 8) treated with the same dose regimen.

There was no difference between the pharmacokinetic parameters obtained at 10, 30, 40, and 60 days after start of treatment during each phase, demonstrating the absence of autoinduction and/or autoinhibition of BNZ elimination processes. For example, when comparing the 30th and 60th days in the acute phase the values were: *C*_max_ (8.10 vs 8.97), *C*_ss_ (5.35 vs 6.84 μg/mL), AUC_0–12_ (64.21 vs 82.04 (μg·h)/mL) Vd/F (19.21 vs 12.24 L) and CL/F (1.31 vs 1.03 L/h), respectively (Table 1). At the same time, in the chronic phase, the values were: *C*_max_ (27.58 vs 16.24), *C*_ss_ (11.85 vs 12.37 μg/mL), AUC_0–12_ (142.25 vs 148.43 (μg·h)/mL), Vd/F (11.84 vs 6.02 L), and CL/F (0.59 vs 0.57 L/h), respectively (Table 2).

**Table 1:** Serum Pharmacokinetic Parameters of Benznidazole during Steady-State Levels at 10, 30, 40, and 60 days after Multiple Oral Doses 3.5 mg/kg b.i.d. Administration (10, 30, 40, and 60 Treatment Days) in Mongrel Dogs, in the Acute State (*n* = 6) of the Experimental Infection with *T. cruzi* Be-78 Strain^a^ ^,^ ^b^

		acute-BNZ			
parameter (unit)	healthy-BNZ	10 days	30 days	40 days	60 days
AUC_0–12_ ((μg·h)/mL)	90.39 (79.65–101.13)	99.94 (79.89–120.98)	64.21 (55.51–77.75)	99.03 (88.97–99.57)	82.04 (77.03–96.52)
CL/F (L/h)	0.84 (0.70–0.98)	0.84 (0.70–1.06)	1.31 (1.08–1.51)	0.85 (0.84–0.95)	1.03 (0.87–1.09)
Vd_ss_/F (L)	17.34 (10.33–24.34)	14.53 (11.83–16.50)	19.21 (12.25–33.09)	14.75 (7.45–22.35)	12.24 (10.77–16.31)
*T*_max_ (h)	3.16 (2.11–4.20)	2.5 (2.00–3.75)	4.00 (3.25–4.00)	2.00 (1.25–3.50)	2.00 (2.00–2.75)
*t*_1/2el_ (h)	11.55 (8.66–17.32)	11.18 (9.71–12.68)	14.59 (7.56–17.61)	11.12 (5.61–18.79)	8.71 (8.16–10.90)
*C*_max_ (μg/mL)	9.70 (8.51–10.88)	11.48 (9.67–16.77)	8.10 (7.36–9.75)	12.35 (11.54–12.77)	8.97 (7.91–10.60)
*C*_ss_ (μg/mL)	7.70 (6.69–8.71)	8.32 (6.66–10.08)	5.35 (4.62–6.48)	8.25 (7.41–8.30)	6.84 (6.42–8.04)
*C*_min_ (μg/mL)	5.86 (4.80–6.91)	5.12 (3.79–5.68)	2.76 (2.50–3.06)	5.58 (3.60–5.74)	4.32 (3.45–4.49)
fluctuation (%)	50.95 (38.64–63.26)	93.23 (76.79–120.39)	107.75 (71.74–126.52)	95.06 (81.40–109.08)	74.86 (66.89–87.78)
*K*_el_ (h^–1^)	0.06 (0.04–0.08)	0.06 (0.05–0.07)	0.05 (0.04–0.09)	0.08 (0.04–0.12)	0.08 (0.06–0.09)

a Data are reported as median (interquartile range).

b *C*_max_: maximum serum concentration; *T*_max_: time to reach *C*_max_; AUC_0–12_: area under the serum concentration vs time curve from 0 to 12 h; *t*_1/2el_: elimination half-life; *C*_ss_: concentration at steady state; Vd_ss_/F: apparent volume of distribution at steady state; CL/F: apparent total clearance at steady state; *K*_el_: constant of elimination; fluctuation: the difference between maximum and minimum plasma concentrations compared with the average serum concentration over a 12-h interval.

**Table 2:** Serum Pharmacokinetic Parameters of Benznidazole during Steady-State Levels at 10, 30, 40, and 60 days after Multiple Oral Doses 3.5 mg/kg b.i.d. Administration (10, 30, 40, and 60 Treatment Days) in Mongrel Dogs, in the Chronic State (*n* = 5) of the Experimental Infection with *T. cruzi* Be-78 Strain^a^ ^,^ ^b^

		chronic-BNZ			
parameter (unit)	healthy-BNZ	10 days	30 days	40 days	60 days
AUC_0–12_ ((μg·h)/mL)	90.39 (79.65–101.13)	169.13 (83.45–190.10)	142.25 (125.92–216.83)	153.08 (126.91–196.43)	148.43 (125.13–171.60)
CL/F (L/h)	0.84 (0.70–0.98)	0.50 (0.44–1.00)	0.59 (0.39–0.67)	0.55 (0.43–0.66)	0.57 (0.49–0.67)
Vd_ss_/F (L)	17.34 (10.33–24.34)	10.80 (8.61–11.94)	11.84 (9.56–13.68)	8.02 (6.32–9.34)	6.02 (5.69–6.10)
*T*_max_ (h)	3.16 (2.11–4.20)	6.00 (4.00–8.00)	2.00 (1.00–2.00)	3.00 (3.00–4.00)	3.00 (2.00–3.00)
*t*_1/2el_ (h)	11.55 (8.66–17.32)	7.98 (7.23–10.87)	16.06 (15.25–17.11)	10.66 (8.46–12.68)	7.91 (6.21–8.06)
*C*_max_ (μg/mL)	9.70 (8.51–10.88)	19.24 (10.64–21.38)	27.58 (18.29–44.82)	17.65 (17.48–22.00)	16.24 (14.80–17.65)
*C*_ss_ (μg/mL)	7.70 (6.69–8.71)	14.09 (6.95–15.84)	11.85 (10.49–18.07)	12.76 (10.5816–16.37)	12.37 (10.43–14.30)
*C*_min_ (μg/mL)	5.86 (4.80–6.91)	8.34 (2.06–10.72)	8.97 (8.15–12.39)	8.10 (4.76–12.81)	5.84 (5.72–9.13)
fluctuation (%)	50.95 (38.64–63.26)	92.47 (92.30–141.44)	78.62 (70.99–422.96)	73.48 (73.03–121.86)	77.33 (59.57–85.06)
*K*_el_ (h^–1^)	0.06 (0.04–0.08)	0.09 (0.07–0.10)	0.043 (0.041–0.045)	0.07 (0.06–0.09)	0.09 (0.09–0.11)

a Data are reported as median (interquartile range).

b *C*_max_: maximum serum concentration; *T*_max_: time to reach *C*_max_; AUC_0–12_: area under the serum concentration vs time curve from 0 to 12 h; *t*_1/2el_: elimination half-life; *C*_ss_: concentration at steady state; Vd_ss_/F: apparent volume of distribution at steady state; CL/F: apparent clearance at steady state; *K*_el_: constant of elimination; fluctuation: difference between maximum and minimum plasma concentrations compared with the average serum concentration over a 12-h interval.

Then, the pharmacokinetic profiles of BNZ obtained at the different treatment times were grouped by phase (acute and chronic) and presented as median (Figures 3 and 4, Table 3). It is possible to observe a similar pharmacokinetic profile between the data from healthy individuals and the acute phase, both differing from the pharmacokinetic profile obtained in the chronic phase of the infection.

**Figure 3:**
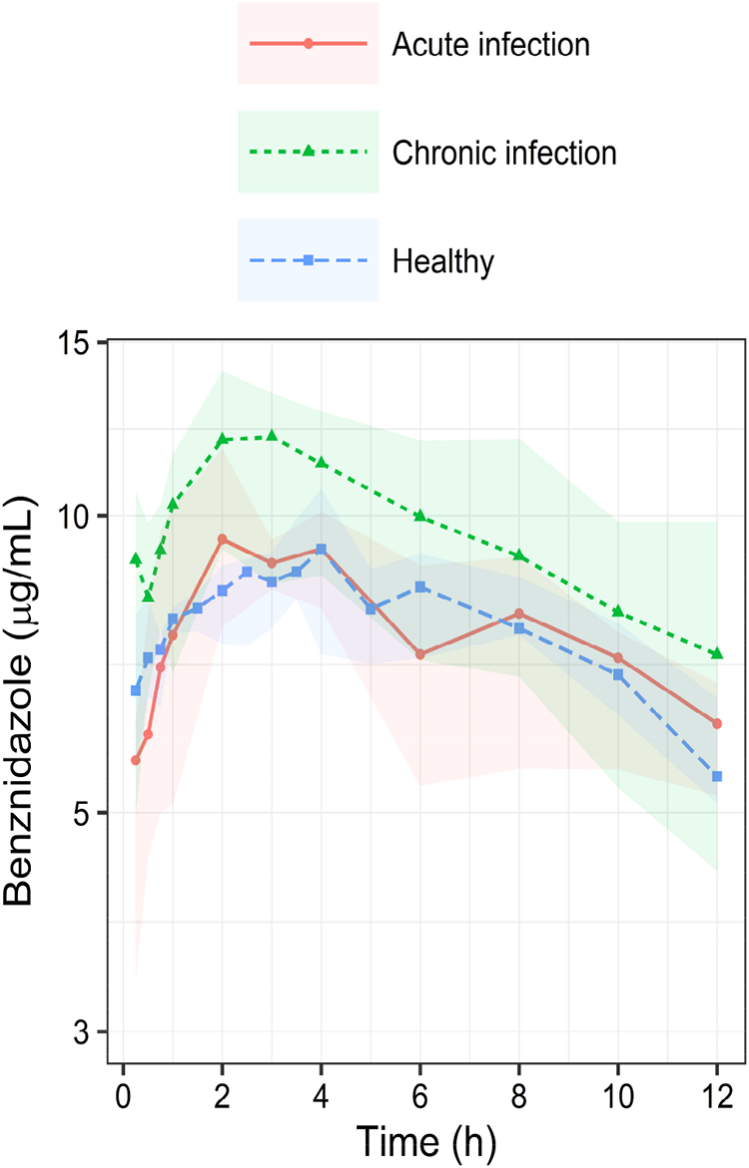
Benznidazole serum concentrations from 0 to 12 h, presented as medians (dots and lines) and 25–75 percentiles intervals (shaded area), in acute (*n* = 6) and chronic (*n* = 5) experimental infection with *T. cruzi* Be-78 strain, and healthy (*n* = 8) dogs (red, green, blue) following treatment of 3.5 mg/kg b.i.d. administration.

**Figure 4:**
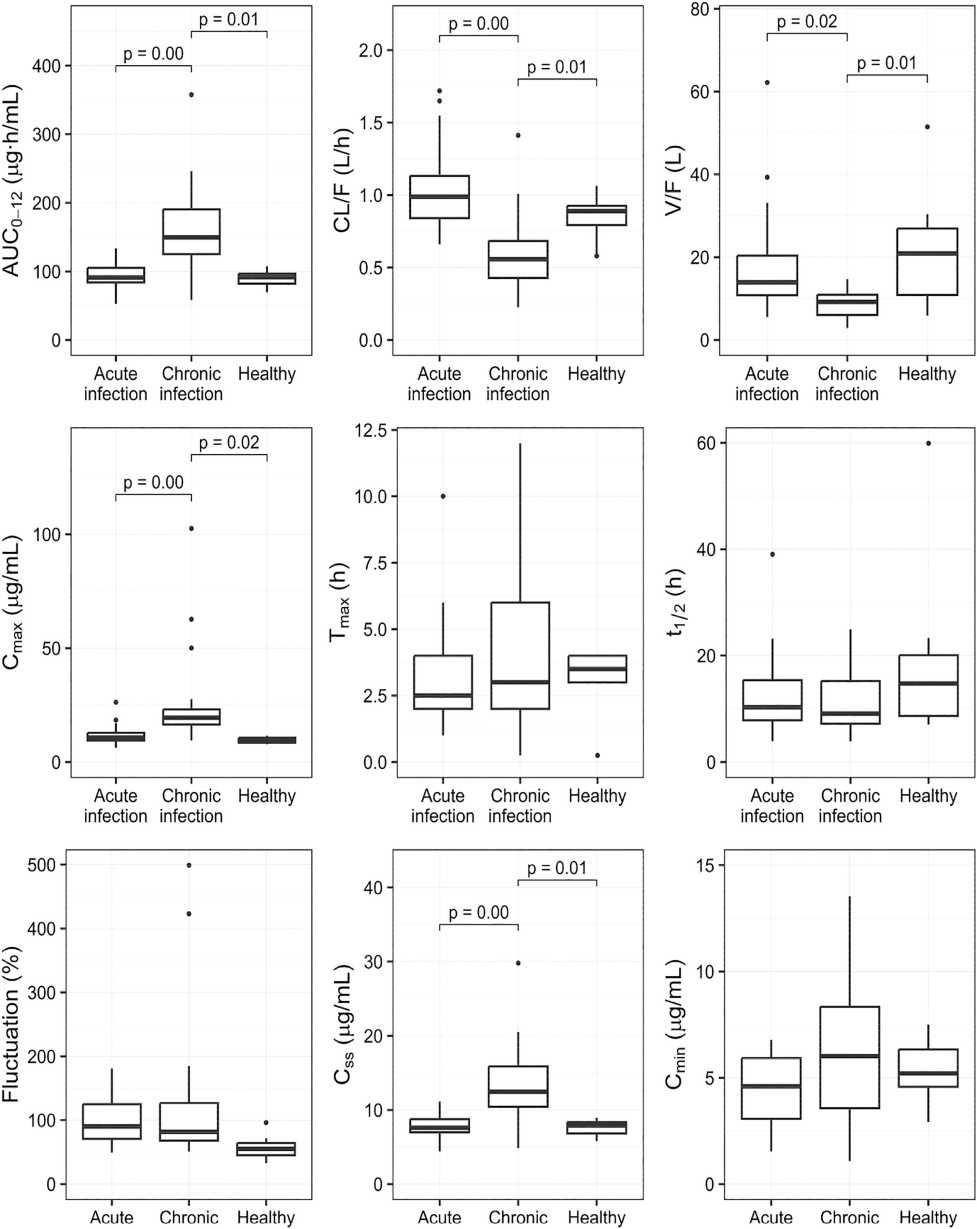
Box-plot of the benznidazole pharmacokinetics parameters in acute and chronic *T. cruzi*-infected and healthy dogs. AUC_0–12_: area under the concentration over time curve. CL/F: apparent clearance, V/F: apparent volume of distribution. *C*_max_: maximum serum concentration achieved, *T*_max_: time to reach *C*_max_. *t*_1/2el_: elimination half-life; fluctuation: difference between maximum and minimum serum concentrations compared with the average serum concentration over a 12-h interval; *C*_ss_: average concentration at steady state. *C*_min_: through concentration. *p*-Values between box-plots assessed by analysis of variance (ANOVA) with Tukey Honest Significant Differences (HSD) for all parameters.

**Table 3:** Serum Pharmacokinetic Parameters of Benznidazole after Multiple Oral Doses 3.5 mg/kg b.i.d. Administration in Mongrel Dogs, in the Acute (*n* = 6) and Chronic State (*n* = 5) of the Experimental Infection with *T. cruzi* Be-78 Strain^a^ ^,^ ^b^

		infected	
parameter (unit)	healthy-BNZ	acute-BNZ	chronic-BNZ
AUC_0–12_ ((μg·h)/mL)	90.39 (79.65–101.13)^#^	84.98 (74.20–99.92)°	150.76 (123.07–196.56)
CL/F (L/h)	0.84 (0.70–0.98)^#^	0.99 (0.84–1.13)°	0.56 (0.43–0.68)
Vd_ss_/F (L)	17.34 (10.33–24.34)^#^	13.92 (10.80–20.36)°	9.20 (6.04–10.93)
*T*_max_ (h)	3.16 (2.11–4.20)	2.50 (2.00–4.00)	3.00 (2.00–6.00)
*t*_1/2el_ (h)	11.55 (8.66–17.32)	10.30 (7.83–15.38)	9.09 (7.19–15.22)
*C*_max_ (μg/mL)	9.70 (8.51–10.88)^#^	10.55 (8.37–12.20)°	17.97 (16.08–23.11)
*C*_ss_ (μg/mL)	7.70 (6.69–8.71)^#^	7.08 (6.18–8.32)°	12.56 (10.26–16.37)
*C*_min_ (μg/mL)	5.86 (4.80–6.91)	4.32 (2.77–5.72)°	8.25 (4.48–11.68)
fluctuation (%)	50.95 (38.64–63.26)	90.26 (70.90–125.10)	81.84 (68.14–126.76)
*K*_el_ (h^–1^)	0.06 (0.04–0.08)	0.07 (0.05–0.09)	0.08 (0.05–0.10)

a Data are reported as medians (interquartile range).

b AUC_0–12_: area under the serum concentration vs time curve from 0 to 12 h; *C*_max_: maximum serum concentration; *T*_max_: time to reach *C*_max_; *t*_1/2el_: elimination half-life; *C*_ss_: concentration at steady state; Vd_ss_/F: apparent volume of distribution at steady state; CL/F: apparent clearance at steady state; *K*_el_: constant of elimination; fluctuation: difference between maximum and minimum serum concentrations compared with the average plasma concentration over a 12-h interval. *p* < 0.05, ANOVA with Tukey Honest significant differences: ^#^ healthy vs chronic-BNZ; ° chronic-BNZ vs acute-BNZ.

It is noted that chronic infection (CHRONIC-BNZ group) with the Be-78 strain of *T. cruzi* increases the values of *C*_max_ (17.97 vs 9.70 μg/mL), *C*_ss_ (12.56 vs 7.70 μg/mL), and AUC_0–12_ (150.76 vs 90.39 (μg·h)/mL) and also reduces Vd_ss_/F (9.20 vs 17.34 L) and CL/F (0.56 vs 0.84 L/h) when compared with healthy animals (HEALTHY-BNZ group), respectively. Comparing the chronic phase (CHRONIC-BNZ group) with the acute phase (ACUTE-BNZ group), respectively, with the chronic disease status also increases the values of *C*_max_ (17.97 vs 10.55 μg/mL), *C*_ss_ (12.56 vs 7.07 μg/mL), *C*_min_ (8.25 vs 4.32 μg/mL), and AUC_0–12_ (150.76 vs 84.98 (μg·h)/mL) and also reduces Vd_ss_/F (9.20 vs 13.92 L) and CL/F (0.56 vs 0.99 L/h) values.

Chronic and acute experimental infection with the Be-78 strain of *T. cruzi* does not alter the rate of drug absorption since the *T*_max_ value, the time at which *C*_max_ is reached, was comparable to healthy dogs. It is also noteworthy that absorption in the dog model is slow (∼3 h), being similar to that observed in humans considering the same route of administration and dose.^4^

After oral administration, the extent of absorption is determined by presystemic elimination in both the intestine and liver, and the higher values observed for AUC_0–12_, *C*_max_, and *C*_ss_ in the experimental chronic infection (CHRONIC-BNZ group) compared to healthy dogs (HEALTHY-BNZ group), indicating that the ChD chronic disease status increases BNZ absorption.

To thoroughly evaluate the benznidazole bioavailability and pharmacokinetics in dogs, additional experiments were conducted to support the present study. Four males and four females of healthy, undefined-breed dogs were evaluated at a single dose of 3 mg/kg administered by intravenous, intraperitoneal, and oral routes. The absolute bioavailability (*F*) value was 100% for both routes, intraperitoneal and oral (Figure S1 and Table S1—Supporting Information). We also observed that the *T*_max_ and absorption constant (*K*_a_) values after intraperitoneal administration are around 2-fold lower and 4-fold higher than oral administration, respectively (Table S1, Supporting Information). Indicating that the slow absorption of benznidazole, despite its high overall bioavailability (*F*% ≈ 100), is likely attributable to its low solubility in the gastrointestinal tract.

Disease-mediated changes can modify the permeability of intestinal barriers. BNZ is considered to have low permeability and a substrate of the efflux protein P-glycoprotein (P-gP).^4,16–19^ Therefore, we hypothesize that the increased oral absorption of BNZ in the chronic phase may be related to the inhibition of this protein, a phenomenon that has been reported in several inflammatory and infectious diseases.^20,21^ However, this correlation can only be confirmed through future studies specifically designed to evaluate P-gp activity using validated substrates such as fexofenadine. Such investigations are already planned by our group to further elucidate the mechanistic relationship among IL-6 levels, P-gp modulation, and BNZ pharmacokinetics.

The apparent volume of distribution at steady state (Vd_ss_/F) of BNZ in healthy dogs is considered low (∼17 L), corroborating with the low Vd values observed in humans.^4,18^ The analysis of this parameter in the chronic phase of experimental infection by the Be-78 strain of *T. cruzi* indicates a reduction in the Vd_ss_/F value (9.20 vs 17.34 L) compared to healthy dogs, respectively. Based on our results and those that also reported low tissue distribution in healthy Swiss mice and those infected with *T. cruzi*,^22,23^ it is suggested that the distribution of BNZ is permeability-limited and is dependent on the activity of membrane transporters.

Clearance is the most important pharmacokinetic parameter to establish a therapeutic regimen, guiding the dose selection to maintain plasma concentrations of the drug at therapeutic levels.^24^ Clearance is directly proportional to the apparent volume of the distribution. This relationship was maintained in our study since the reduction in Vd_ss_ values was accompanied by a decrease in CL/F values (0.56 vs 0.84 L/h) in the chronic infection group compared to healthy dogs, respectively.

Thus, it is noted that experimental chronic infection with the Be-78 strain of *T. cruzi* alters the pharmacokinetic parameters of BNZ, increasing the absorption of BNZ in addition to decreasing the volume of distribution and clearance in the dog model. Therefore, considering a potential change in the BNZ dose regimen in chronically infected patients may be necessary. In this context, leveraging pharmacometric tools becomes crucial to validate dog models as a standard model for predicting the pharmacokinetics of BNZ in humans.

The pharmacokinetics of BNZ could be different between chronic ChD patients and healthy individuals, thus, an appropriate animal model must demonstrate this difference to generate predictable data to translate to humans.^14,15,25,26^ However, contrary to our results, a study carried out in BALB/C mice infected with the CL Brener strain of *T. cruzi* and treated with a dose of 100 mg/kg of BNZ demonstrated that the chronic phase of infection does not alter the pharmacokinetic parameters of this drug.^27^ This conflicting result can be attributed to several factors, including the use of different strains of mice, variations in species, and duration of chronic infection. underscores the complexity of ChD, where outcomes of infection and responses to treatment are shaped by intricate interactions between host genetics and physiology as well as parasite genetics. Work carried out by our group, through a scale-up of mice^25,26^ and dogs experimental models, has demonstrated agreement in results when using the Be-78 *T. cruzi* strain. Therefore, the Swiss mouse and the mongrel dog may be appropriate models to evaluate the impact of chronic infection on pharmacokinetics, being of great translational value for better understanding pharmacotherapy in ChD.

The finding that experimental chronic infection alters the pharmacokinetics of BNZ in this study could partly explain the therapeutic failure in the chronic phase of ChD, making it necessary to understand the role of the inflammatory process caused by the infection in the observed pharmacokinetic changes.

In the present work, an analysis of the inflammatory response in the different phases of the disease was proposed through serum measurement of the cytokines IL-6, IFN-γ, TNF-α, and IL-10 (Figure 5).

**Figure 5:**
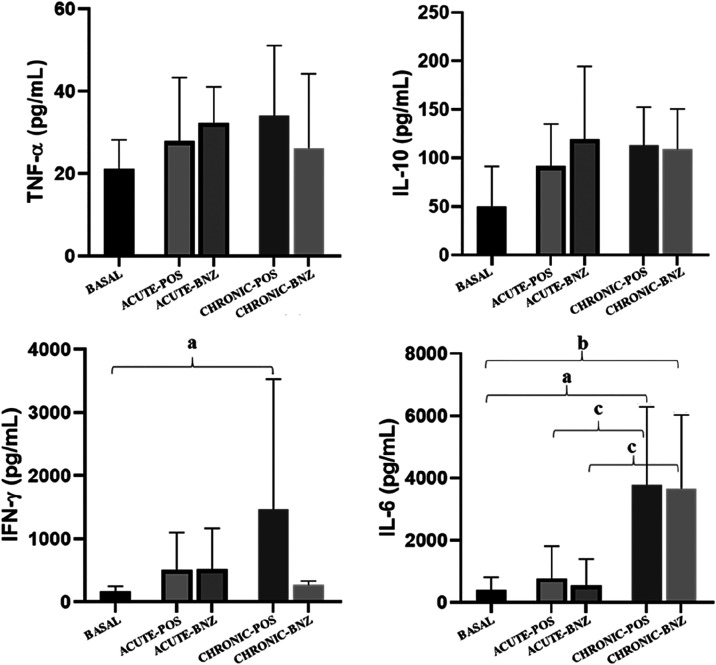
Cytokines panel (IFN-γ, IL-10, TNF-α, and IL-6) evaluated at basal state (BASAL), infection without treatment (ACUTE-POS) (*n* = 4), and treated with oral benzonidazole (BNZ) 3.5 mg/kg b.i.d. administration (ACUTE-BNZ) (*n* = 6) in the acute phase and infection without treatment (CHRONIC-POS) (*n* = 4) and treated in the chronic phase (CHRONIC-BNZ) (*n* = 5) during the experimental infection with *T. cruzi* Be-78 strain. Statistical comparisons are indicated in the figure as follows: (a) CHRONIC-POS vs Basal, *p* < 0.05; (b) CHRONIC-BNZ vs Basal, *p* < 0.05; (c) ACUTE-POS vs CHRONIC-POS and ACUTE-BNZ vs CHRONIC-BNZ, *p* < 0.05.

An inflammatory status is a common characteristic of the changes observed in the cardiac and/or digestive tissues in patients with ChD.^28^ There is a strong possibility that various cytokines and chemokines apart from the ones that we already investigated are regulated in ChD, such as IFN-γ, TNF-α, IL-12, IL-4, SCGF β, CXCL9, IL-10, and IL-6, could be used, alone or in combination, as diagnostic and prognostic markers in the future. They play an important role in the pathogenesis and progression of the acute and chronic diseases.^27–30^

The cytokines IL-6, IFN-γ, and TNF-α are related to the progression of the disease, playing a proinflammatory role, and the cytokine IL-10 acts to contain the inflammatory process, relating to host protection. Therefore, the pro-inflammatory cytokine generation due to the activation of innate mechanisms is important to control parasitemia. However, this scenario of exacerbated inflammation generates tissue damage and the role of IL-10 is considered important because it is an immunomodulatory cytokine produced by T lymphocytes and monocytes.^31^

The analysis of the cytokine profile in the experimental infection of the dog model with the Be-78 strain of *T. cruzi* indicates that during the acute and chronic phases, there is an increase in the cytokines IL-6, IFN-γ, IL-10, and TNF-α, to baseline, in animals before infection. This data aligns with the findings reported in a study by Sousa et al.,^32^ which analyzed plasma samples from 176 patients infected with *T. cruzi* and 24 healthy individuals, being demonstrated that the expression of plasma inflammatory cytokines, such as IFN-γ, TNF-α, and IL-6, was higher in those with the cardiac form of the disease.^32^ The strain used in the experimental protocol of the present study was Be-78 that is a strain presenting a predominance of broad forms and tropism for smooth, skeletal, and cardiac muscle cells.

Previous studies indicate that in addition to its trypanocidal activity, BNZ also affects the synthesis of biological markers involved in inflammation.^33^ However, our results indicate that there was no change in the serum concentrations of the cytokines IL-10 and TNF-α when comparing infected and untreated animals and those treated with a multiple oral dose of 3.5 mg BNZ/kg/12 h during the acute and chronic phases of the infection (Figure 5). TNF-α is an inflammatory marker considered a predictor of mortality^31^ and is generally increased in the chronic phase compared to the acute phase of the disease, especially in infections with strains with greater tropism for cardiomyocytes, e.g., Be-78.^32^ A possible explanation for our finding is that the animals used in the study were chronically infected only for 1 year; therefore, the TNF-α concentrations did not exhibit significant differences compared to the basal state. IL-10, on the other hand, is produced by monocytes and is involved in tissue repair^34^ and is typically increased regardless of the stage of the disease, a situation also observed in the present study.

Some studies have described that one of the factors that can potentially influence the effectiveness of ChD treatment is the possible cooperative action between the effects of drugs and the host’s immune response.^35^ In our work, we observed a reduction in the production of IFN-γ to almost basal levels when the animals were treated during the chronic phase of infection, suggesting that BNZ may have acted cooperatively with the immune system, being able to modify the inflammatory response during the chronic phase of the infection.

The high production of IL-6 in the acute and chronic phases may be associated with pathological aspects of the disease. It is known that *T. cruzi* infection in animal models increases serum and tissue IL-6 levels, which is induced during the parasitemia increasing in the acute period of ChD.^36,37^ For Saavedra et al.,^38^ the main inducer of this increase in IL-6 levels in *T. cruzi* infection is the transialidase enzyme produced by the parasite itself. In this sense, it is possible to hypothesize, based on our results, that the higher IL-6 levels observed in the chronic phase of the disease may be associated with the persistence of infection in these animals, as confirmed by the parasite load presented in Figure S2 (Supporting Information).

It is known that serum concentrations of IL-6, a pro-inflammatory mediator, range from ∼1 to 10 pg/mL in healthy individuals, but can be increased to between ∼10 and 1500 pg/mL in patient populations with inflammation, such as rheumatoid arthritis, or after surgical trauma, infections, and organ transplantation.^36–40^

Our results showed that IL-6 levels had a significant increase (up to 7-fold) in their serum concentrations in dogs in the chronic phase compared to the acute phase, even when treatment with BNZ is administered. It is worth noting that the phenomenon of inhibition of enzymes and proteins related to the kinetic disposition of drugs is directly related to a significant increase in the serum concentration of cytokines, especially IL-6.^36,37^ Lanchote et al.^39^ proposed that concentrations higher than 50 pg/mL of IL-6 are necessary for CYP3A and CYP2C19 downregulation in patients with visceral leishmaniasis.^39^ More recently, it was demonstrated that low concentrations of IL-6 in a group of chagasic patients (1.5–5.1 pg/mL) were not sufficient to cause a downregulation in UGT and therefore impact the pharmacokinetics of nebivolol and its metabolites.^41^

In silico, in vitro, and clinical studies have demonstrated that inflammatory and/or infectious diseases such as cancer,^42^ heart failure,^43^ multiple sclerosis,^44^ psoriasis,^45^ malaria,^46^ and visceral leishmaniasis^39^ can alter the expression and activity of CYP450 enzymes and the efflux transporter protein P-gP most likely due to the release of pro-inflammatory cytokines. For ChD, this correlation is not yet well established, and the present work provides the opportunity to fill this gap in the literature by demonstrating that experimental chronic infection with the Be-78 strain of *T. cruzi* does have an impact on the pharmacokinetics of BNZ in the dog model. In this context, it is pertinent to relate the high mean serum concentrations of IL-6 observed in the chronic phase (>3000 pg/mL) with the changes in the pharmacokinetic parameters of BNZ in the dog model. Interestingly, mean serum concentrations of up to ∼770 pg/mL in dogs in the acute phase of the disease were not able to alter the kinetic disposition of BNZ.

It is important to note that the dosing regimen for BNZ was originally established based on studies conducted in healthy individuals. However, according to the FDA,^16^ the pharmacokinetics of BNZ may differ significantly in chronically infected individuals. Our data support this hypothesis, as the marked increase in IL-6 levels observed during the chronic phase suggests persistent inflammation, which may be associated with the reduced efficacy of treatment at this stage of the disease.

Longitudinal studies in patients with inflammatory conditions have shown that, following the resolution of inflammation and infection, enzyme levels and membrane transporters return to baseline, thereby normalizing the pharmacokinetic profile of drugs and contributing to treatment efficacy.^47–51^ However, the data presented here indicate that in the context of chronic Chagas disease infection, this normalization does not occur, potentially compromising the effectiveness of BNZ.

As BNZ is considered a substrate of the P-gP efflux protein,^4,19,21,52,53^ the changes observed in pharmacokinetics may be associated with the inhibition of this transporter due to the pronounced increase in IL-6 observed during the chronic phase of the infection.

Among the limitations of this study is the lack of extended follow-up during the chronic phase after treatment, which prevents assessment of whether inflammation decreases over time and potentially impacts BNZ pharmacokinetics. In addition, different dosing regimens or frequencies were not evaluated, which could be addressed in future pharmacometric studies. The activity of the P-glycoprotein (P-gp) transporter, which may influence the drug’s bioavailability, was also not investigated, and future in vivo studies in ChD can contribute to confirming this hypothesis.

## Methods

### Animals and Ethics

Twenty-seven mixed-breed dogs (13 males, 14 females), aged 4–10 months and weighing 10–30 kg, were used. Animals were housed at the Animal Science Center of the Federal University of Ouro Preto (CCA/UFOP) under the CONCEA guidelines. The study was approved by the Animal Experimentation Ethics Committee of UFOP (protocol 2017/38), and veterinary monitoring and routine care were provided throughout the study.

### Sample Size Calculation

Sample size was calculated by using PS: Power and Sample Size Calculation v3.1.2, based on BNZ pharmacokinetics in healthy dogs.^54^ The study was powered to detect an ≥30% increase in BNZ AUC due to cytokines from acute or chronic *T. cruzi* infection, with α = 0.05 and 80% power. To account for potential mortality, six dogs were included per treatment group.

### Inoculum and Infection

The *T. cruzi* Berenice-78 strain inoculum was obtained from Swiss mice at peak parasitemia and standardized using sterile phosphate-buffered saline (PBS) (pH 7.2). Dogs were infected intraperitoneally with 2000 trypomastigotes/kg. Infection was confirmed by daily parasitemia monitoring until five consecutive negative tests were obtained.^55^ Dogs were observed for clinical signs and behavioral changes.

### Experimental Protocol

The study design is depicted in Figure 1. The 27 dogs were randomly divided into the following groups: acute infection state treated with BNZ (ACUTE-BNZ) (*n* = 6), chronic infection state treated with BNZ (CHRONIC-BNZ) (*n* = 5), acute (ACUTE-POS) (*n* = 4), and chronic (CHRONIC-POS) (*n* = 4) positive controls (not treated with BNZ), and a healthy group treated with BNZ (HEALTHY-BNZ) (*n* = 8). The ChD experimental groups were evaluated at (1) basal state for cytokines panel evaluation (IL-6, IFN-γ, IL-10, and TNF-α); (2) during infection without treatment for cytokines; (3) during BNZ steady-state levels at 10, 30, 40, and 60 days after start of treatment with oral BNZ 3.5 mg/kg b.i.d. administration for cytokines and/or BNZ pharmacokinetics evaluation; (4) 30 days after BNZ treatment end for cytokines evaluation.

Acute and chronic phases were defined as ≤1 month and ≥12 months postinfection, respectively. Washout periods of 3–5 days were applied between experimental occasions.

### Pharmacokinetic Study

The pharmacokinetic study was conducted during occasion 3. The ACUTE-BNZ group received treatment with BNZ from the first day of patent parasitemia, confirmed by a fresh blood test, and the CHRONIC-BNZ group received treatment with BNZ after 12 months of infection.

BNZ tablets (100 mg, LAFEPE) were compounded into capsules individualized per dog weight. Capsules were administered orally with palatabilizers; ingestion was confirmed by gentle stimulation of the swallowing. Dogs were not fasted or water-restricted. Serial blood samples (0, 0.25, 0.5, 0.75, 1, 2, 3, 4, 6, 8, 10, and 12 h) were collected during steady-state treatment.

### Parasitological Evaluation

Daily fresh-blood examinations were performed from day 10 postinfection until five consecutive negatives.^55^ Trypomastigotes were counted microscopically to confirm the infection stage.

### Sample Preparation and Bioanalysis

The blood collection method used throughout the study was cephalic vein puncture. Serum was obtained by centrifugation and stored at −80 °C. BNZ concentrations were quantified by high-performance liquid chromatography with diode array detection (HPLC-DAD). The HPLC system consisted of chromatography equipment from Shimadzu (Kyoto, Japan) equipped with an LC-20AT pump, an autoinjector model SIL-20A HT, an oven CTO-20A, and a controller system model SCL 20A, equipped with a DAD detector model SPD-M20A operating at 324 nm. An analytical C18 column (Gemini-NXVR, Phenomenex, Torrance, CA; 150 mm × 4.6 mm, 5 μm) and a C18 column guard (model AJ0-7597VR, Phenomenex, Torrance, CA; 4 mm × 3 mm) were maintained at 40 °C. The mobile phase was composed of a mixture of water and acetonitrile (ACN) (65:35, v/v) at 1 mL/min, 20 μL injection, and detection at 324 nm. The method was validated per EMA 2011 guidelines: linearity 0.1–100 μg/mL (*r* ^2^ > 0.99), precision 3.14–10.41%, accuracy 1.0–10.5%, and stability ≤15% coefficient of variation (CV).

### Standard Solutions and Reagents

Standard solutions and reagents benznidazole (BNZ) stock solution (99%, Sigma-Aldrich, St. Louis, MO) was prepared at 4000 μg/mL in acetonitrile (ACN). Working solutions were prepared by serial dilution (2–2000 μg/mL) in ACN. Omeprazole (internal standard, 99%, Sigma-Aldrich) stock solution was prepared at 2000 μg/mL in ACN and diluted to 200 μg/mL. All solutions were stored at −20 °C. Chromatography-grade solvents were obtained from J.T. Baker (Fairfield), and ultrapure water was obtained using a Milli-Q Direct 8 system (Millipore, Molsheim, France).

### Pharmacokinetic and Statistical Analysis

PK parameters (*C*_max_, *T*_max_, *C*_min_, AUC_0–12_, *K*_el_, *t*_1/2_, *C*_ss_, CL/F, and Vd/F) were calculated using Phoenix WinNonLin v7.0. Statistical analyses were conducted in R v3.4.31 using ANOVA with Tukey HSD for comparisons across times and groups; significance was set at *p* < 0.05.

### Sample Preparation

Blank serum samples were obtained from the same animals enrolled in this study. Blood was collected on different days and at various time points from untreated dogs, and the resulting sera were pooled to prepare a blank matrix (blank serum pool).

For sample processing, 100 μL of serum was mixed with 5 μL of the internal standard solution and 500 μL of acetonitrile. The mixture was vortexed for 10 min to ensure complete protein precipitation, followed by centrifugation to separate the organic phase. The supernatant was then evaporated to dryness under reduced pressure using a rotary evaporator. The residue was reconstituted in 100 μL of the mobile phase, vortexed, and centrifuged again. Finally, 85 μL of the resulting supernatant was transferred to vials and 20 μL was injected into the HPLC system for analysis.

### Validation of the Bioanalytical Method for Benznidazole Analysis

The bioanalytical method developed for quantifying benznidazole (BNZ) in dog serum by HPLC was validated according to the European Medicines Agency^21^ guidelines. The method demonstrated selectivity with no significant interference from biological matrices or carryover effects from previously analyzed samples. Calibration curves were constructed in triplicate using 100 μL aliquots of blank serum spiked with 5 μL of BNZ standard and internal standard solutions, over the concentration range of 0.1–100 μg/mL. The method exhibited excellent linearity (*r* ^2^ > 0.99) within this range.

Intra- and interassay precision ranged from 3.14 to 10.41%, and accuracy varied from 1.00 to 10.50%, confirming the method’s reliability. Sample stability was demonstrated by coefficients of variation and inaccuracy values ≤15% under short-term (4 h at room temperature), postprocessing (24 h at 21 ± 1 °C), and three freeze–thaw cycle conditions, when compared to freshly prepared samples.

### Pharmacokinetic and Statistical Analysis

Dogs acutely (*n* = 6) and chronically (*n* = 5) infected with the *T. cruzi* Berenice-78 strain were treated with benznidazole (BNZ) at a dose of 3.5 mg/kg twice daily (b.i.d.) for 60 consecutive days. Blood samples were collected 10, 30, 40, and 60 days after the beginning of treatment from both groups. At each collection point, serial samples were obtained over a 12 h dosing interval (0–12 h). Serum concentrations of BNZ were determined using the validated HPLC-DAD method described previously.

Pharmacokinetic parameters were calculated using Phoenix WinNonlin version 7.0 software (Pharsight, Certara). The following parameters were determined: *C*_max_ (maximum observed concentration), *T*_max_ (time of *C*_max_), *C*_min_ (minimum observed concentration), AUC_0–12_ (area under the observed concentration over time data calculated by trapezoidal rule), *K*_el_ (first-order rate constant of the terminal portion of the concentration over time curve), *t*_1/2el_ (elimination half-life, *t*_1/2el_ = ln(2)/*K*_el_), *C*_ss_ (concentration at steady-state, *C*_ss_ = AUC_0–12_/12), CL/F (apparent total clearance at steady state, CL/F = dose/AUC_0–12_); Vd/F (apparent volume of distribution at steady state, Vd/F = CL/F/*K*_el_).

Statistical summaries, tests, and graphics were carried out using the software R version 3.4.3. A 5% significance level was considered to compare across treatment times (10, 30, 40, and 60 days in acute and chronic *T. cruzi*-infection) and groups (data grouped by phase—acute and chronic) by ANOVA with Tukey Honest Significant Differences for all parameters.

### Serum Cytokine Analysis Using Enzyme-Linked Immunosorbent Assay (ELISA)

The concentrations of IL-6, IFN-γ, IL-10, and TNF-α in serum samples were determined using commercial ELISA kits (R&D Systems, Minneapolis, MN), according to the manufacturer’s instructions. Briefly, 96-well MaxiSorp plates (NUNC) were coated overnight at room temperature with the capture antibodies diluted in sterile PBS (pH 7.4). After washing with PBS–Tween 20 solution, plates were blocked with 1% bovine serum albumin (BSA) in PBS for 1 h at room temperature.

Samples and standards were then added to the wells and incubated for 2 h at room temperature, followed by washing and incubation with the biotinylated detection antibody for 2 h. After additional washing steps, plates were incubated with streptavidin–horseradish peroxidase (HRP) conjugate for 20 min, protected from light. The reaction was developed using 3,3′,5,5′-tetramethylbenzidine (TMB) substrate and stopped with 1 M H_2_SO_4_. Absorbance was measured at 450 nm by using a microplate reader.

Cytokine concentrations were determined from standard curves generated for each analyte. Statistical analyses were performed using GraphPad Prism 8. Normality was assessed by the Kolmogorov–Smirnov test. Nonparametric data were analyzed using the Mann–Whitney test (acute vs chronic phase) or the Kruskal–Wallis test followed by Dunn’s post hoc test (comparison among groups within the same phase). Results were considered significant for *p* < 0.05.

### Ethical Approval

The research protocol (No. 2017/38) was approved by the Animal Experimentation Ethics Committee of the Federal University of Ouro Preto (CEUA/UFOP, Brazil) and was forwarded to the local UFOP Animal Science Center (CCA/UFOP). Twenty-seven mixed-breed dogs (13 males and 14 females; 4–10 months old; 10–30 kg) were included. All procedures followed CONCEA (Brazil) guidelines with measures to minimize suffering and adherence to the 3Rs (Replacement, Reduction, and Refinement).

## Conclusion

The present study evaluated the impact of experimental acute and chronic ChD infection by the Be-78 strain of *T. cruzi* on the pharmacokinetics of BNZ in dogs. Acute experimental infection with the Berenice-78 strain of *T. cruzi* does not alter the BNZ pharmacokinetic parameters after multiple dose administration in mongrel dogs. Experimental chronic infection with the Berenice-78 strain of *T. cruzi* increases the values of *C*_max_, *C*_ss_, and AUC_0–12_ and also reduces Vd_ss_/F and CL/F when compared to healthy mongrel dogs.

Experimental acute and chronic infection with the Berenice-78 strain of *T. cruzi* increases serum levels of mediators of the inflammatory process, with IL-6 being the most pronounced cytokine, especially in the chronic phase of infection with up to 7-fold increase compared to the basal state.

The present study is the first scientific evidence that chronic experimental infection with the Berenice-78 strain of *T. cruzi* alters the pharmacokinetics of BNZ in dogs, with an observed increase in the pro-inflammatory cytokine IL-6. The present study indicates that the dog model corroborates to translate benznidazole pharmacokinetics in the preclinical phase to the clinical Chagas disease study. In this context, the use of pharmacometric tools, such as physiologically based pharmacokinetic modeling approach, is important to validate the dog as a standard model to predict the pharmacokinetics of benznidazole and other anti-*T. cruzi* drug candidates in humans.

## Supplementary Material



## Data Availability

The data sets obtained and/or analyzed during the current study are available from the corresponding author on reasonable request.

## Notes

Detailed experimental procedures and data related to the bioavailability study (benznidazole serum concentration–time profiles (Figure S1); pharmacokinetic parameters in healthy dogs (Table S1)) and parasite load assessment by qPCR (Figure S2) (PDF)

## Abbreviations

ACN: acetonitrile
AUC: area under the curve
AUC_0–12_: area under the concentration–time curve from 0 to 12 h
Be-78: Berenice-78 strain of *Trypanosoma cruzi*
BNZ: benznidazole
BSA: bovine serum albumin
ChD: Chagas disease
CL/F: apparent clearance at steady-state
*C*_max_: maximum observed concentration
*C*_min_: minimum observed concentration
*C*_ss_: concentration at steady-state
EMA: European Medicines Agency
*F* (absolute bioavailability): fraction of drug absorbed systemically
FDA: U.S. Food and Drug Administration
HPLC: high-performance liquid chromatography
HPLC-DAD: HPLC with diode array detector
IFN-γ: interferon γ
IL-10: interleukin 10
IL-6: interleukin 6
*K*_a_: absorption rate constant
*K*_el_: elimination rate constant
*N*_m_: number of measurements
PBS: phosphate-buffered saline
PBS-Tween: PBS with Tween 20
P-gP: P-glycoprotein
*t*_1/2el_: elimination half-life
*T*_max_: time to maximum concentration
TNF-α: tumor necrosis factor α
Vdss/F: apparent volume of distribution at steady-state
