# Supplementary material for: Influence of Chagas Disease on the Pharmacokinetics of Benznidazole in the Dog Model
Source: ACS Pharmacol Transl Sci. 2026 Feb 18;9(3):596–607. doi: 10.1021/acsptsci.5c00571 (PMC12993771; doi:10.1021/acsptsci.5c00571)
Supplement: Supplementary file 1 [file pt5c00571_si_001.pdf]

Supporting Information

Influence of Chagas disease on the pharmacokinetics of benznidazole in the dog model

Lorena Cera Bandeira[1]*, Leonardo Pinto[1], Fernanda de Lima Moreira[2], Glauco Henrique Balthazar Nardotto[3], Luciana da Fonseca Medeiros[1], Kátia Fonseca[1], Paula Melo de Abreu Vieira[1], Cláudia Martins Carneiro[1,4]

[1]Laboratory of Immunopathology, Nucleus of Biological Sciences Research, Federal University of Ouro Preto, Ouro Preto, Minas Gerais, Brazil, 35400-000;

[2]Laboratory of Pharmacometrics, Faculty of Pharmacy, Federal University of Rio de Janeiro, Rio de Janeiro, Rio de Janeiro, Brazil, 21941-599;

[3]Bioanalytics, Metabolomics & Pharmacokinetics resources (BMPK), Roswell Park Comprehensive Cancer Center, Buffalo, NY, USA,14263.

[4]Department of Clinical Analysis, School of Pharmacy, Federal University of Ouro Preto, Ouro Preto, Minas Gerais, Brazil, 35400-000.

(*)Corresponding author:
Lorena Cera Bandeira — lorena.bandeira@ufop.edu.br

Table of Contents

Additional Methodological Details (S-2)
  Bioavailability study in healthy animals (S-2)
  Parasitic load quantification by qPCR (S-4)

Supporting Figures (S-6)
  Figure S1. Benznidazole bioavailability study (S-5)
  Figure S2. Parasite load assessment by qPCR (S-6)

Supporting Table (S-7)
  Table S1. Pharmacokinetic parameters in healthy dogs (S-7)

References (S-8)

Additional Methodological DetailsBioavailability study in healthy animals

Experimental Protocol

Four male and four female mixed-breed dogs were used in a randomized three-way crossover design to evaluate the pharmacokinetics of benznidazole (BNZ) after intravenous (IV), intraperitoneal (IP), and oral (VO) administration. Each animal received all three treatments, with a washout period of 3–5 days between each phase. The experimental design also accounted for the influence of circadian rhythm on drug pharmacokinetics. Clinical, hematological, and biochemical parameters were monitored before, during, and after each treatment to assess the animals' health status.

For oral administration, BNZ capsules were prepared with individualized doses adjusted according to body weight. For IV and IP routes, BNZ was administered at 3.5 mg/kg in a vehicle composed of polyethylene glycol 200 and dimethylacetamide (70:30, v/v).

Serial blood samples were collected from the cephalic vein using non-heparinized syringes at the following time points: 0 (predose), 0.25, 0.5, 1, 1.5, 2, 2.5, 3, 4, 6, 8, 12, 16, 24, 36, and 48 hours after administration (performed at either 7 a.m. or 7 p.m.). Serum was obtained by centrifugation and stored at –70 °C until analysis.

The Ethics Committee on Animal Experimentation of the Federal University of Ouro Preto, Minas Gerais, Brazil approved the protocol (number 2016/37). Eight healthy mongrel dogs weighting 20 to 24 kg (mean of 22 kg) were created and maintained in the kennel of Centro de Ciência Animal (UFOP).

Benznidazole analysis in serum using HPLC and bioanalytical validation

BNZ concentrations in serum were determined by a validated high-performance liquid chromatography method with diode array detection (HPLC-DAD).

A stock solution of benznidazole (≥99%, Sigma-Aldrich, St. Louis, MO, USA) was prepared at 4000 µg/mL in acetonitrile and further diluted to concentrations ranging from 2 to 2000 µg/mL. Omeprazole (≥99%, Sigma-Aldrich, St. Louis, MO, USA) was used as the internal standard (IS), prepared at 2000 µg/mL in acetonitrile and diluted to 200 µg/mL in methanol. All stock solutions were stored at –20 °C.

For sample preparation, 100 µL of serum was mixed with 5 µL of IS solution and 500 µL of acetonitrile. After extraction, the organic phase was evaporated, and the residue was reconstituted in 100 µL of the mobile phase.

Chromatographic separation was achieved on a Shimadzu Prominence LC-20AT system equipped with a SPD-M20A DAD detector set at 324 nm. The analytical column was a Gemini-NX C18 (150 × 4.6 mm, 5 µm; Phenomenex, Torrance, CA, USA) with a corresponding C18 guard column (4 × 3 mm, Phenomenex), maintained at 40 °C. The mobile phase consisted of water and acetonitrile (65:35, v/v), delivered isocratically at 1.0 mL/min. The injection volume was 20 µL, and total run time was 5 min.

The bioanalytical method was validated according to EMA guidelines[1] and demonstrated adequate selectivity, linearity, accuracy, and precision, with no interference from endogenous serum components or carryover effects.

Bioavailability and Statistical analysis

The area under the serum concentration over time from 0 to 48 hours (AUC_{0-48}) was assessed by the trapezoidal rule[2] from the BNZ observed plasma concentrations for the intraperitoneal (IP), oral (VO) and intravenous (IV) administrations. The benznidazole

bioavailability of oral (Fvo) and intraperitoneal BNZ administration were calculated by Fvo = $AUC_{0\text{-}48\ VO} \cdot dose_{IV} / AUC_{0\text{-}48\ IV} \cdot dose_{VO}$, and $F_{IP} = AUC_{0\text{-}48\ IP} \cdot dose_{IV} / AUC_{0\text{-}48\ VI} \cdot dose_{IP}$

All parameters were obtained individually and then summarized in R version 4.5.1[3].

Parasitic Load Quantification by qPCR

Genomic DNA was extracted from 100 µL of whole blood using the DNeasy® Blood and Tissue Kit (Qiagen®, Hilden, Germany) according to the manufacturer's instructions. Briefly, samples were incubated with 20 µL of Proteinase K and 200 µL of Buffer AL at 56 °C for 10 min, followed by ethanol precipitation and column-based purification. DNA was eluted in 30 µL of Buffer AE, quantified using a NanoDrop 2000 spectrophotometer (Thermo Scientific, USA), and stored at −20 °C until analysis.

A standard curve was prepared using epimastigotes of *T. cruzi* Y strain cultured in LIT medium, adjusted to 10^6 parasites/mL, and serially diluted tenfold to generate seven points (10^6–1 parasites). qPCR reactions were performed in 96-well optical plates (MicroAmp®, Applied Biosystems, USA) using SYBR Green PCR Master Mix (Applied Biosystems, USA) in a total reaction volume of 10 µL per well, containing 10 ng of genomic DNA and 2 µL of specific primers: TCZ-F 5'-GCTCTTGCCCACAMGGGTGC-3' and TCZ-R 5'-CCAAGCAGCGGATAGTTCAGG-3', amplifying a 182 bp fragment. GAPDH was used as an internal control to assess DNA integrity, with primers 5'-TTCCACGGCACAGTCAAG-3' and 5'-ACTCAGCACCAGCATCAC-3' amplifying a 115 bp fragment. Thermal cycling consisted of initial denaturation at 95 °C for 10 min, followed by 40 cycles at 95 °C for 15 s and 63 °C for 1 min.

Parasite load was evaluated in the acute infection phase at 10, 70, and 100 days post-infection, and in the chronic infection phase at 300, 360, and 390 days post-infection. Quantification of parasite DNA in each sample was determined by interpolation from the standard curve. Samples with Ct values above the lowest point of the standard curve were considered negative. Results are expressed as parasites/mL of blood.

Statistical Analysis: Parasite load data were analyzed using non-parametric methods due to their skewed distribution. Differences between groups and time points were assessed using the Kruskal–Wallis test followed by Dunn's post hoc test. Differences with $p < 0.05$ were considered statistically significant.

Supporting Figures

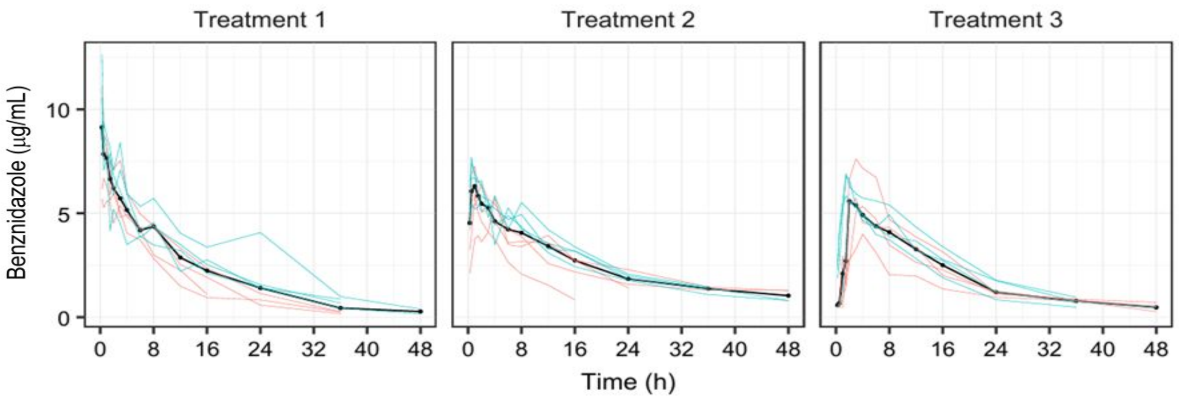

Figure S1: Benznidazole serum concentration over time from 0 to 48 hours time profile of benznidazole after oral administration in healthy dogs (n = 8). Treatment 1, single intravenous dose 3.5 mg/Kg; Treatment 2, single intraperitoneal dose 3.5 mg/Kg; Treatment 3, single oral dose 3.5 mg/Kg.

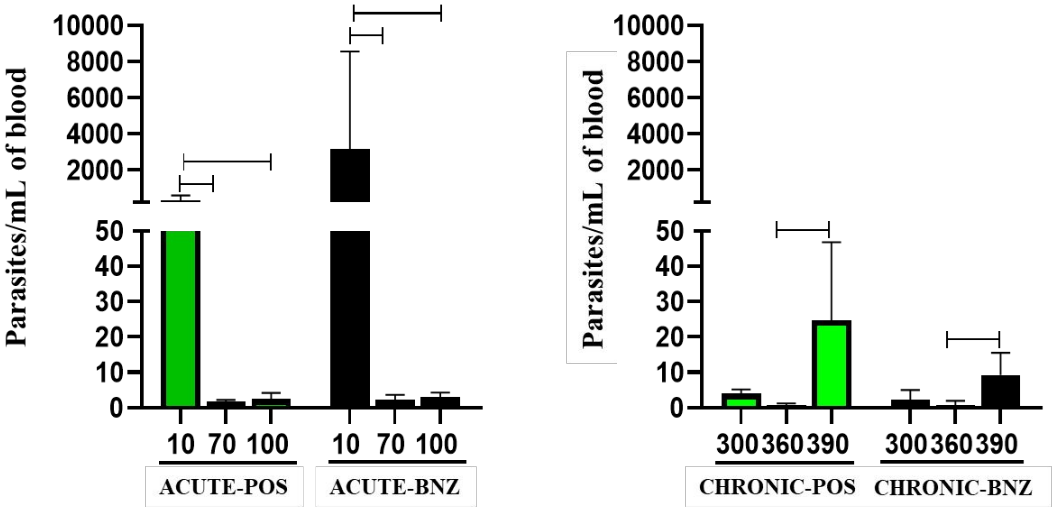

Figure S2: Parasitic load quantified by qPCR in experimentally infected SRD dogs with T. cruzi Be-78 strain. Groups: infected and untreated in acute (ACUTE-POS) and chronic infection (CHRONIC-POS) and infected treated with oral Benzonidazole (BNZ) 3.5mg/Kg b.i.d administration in acute (ACUTE-BNZ) and chronic infection (CHRONIC-BNZ). Parasite load was evaluated at 10, 70, and 100 days post-infection for the acute infection and at 300, 360, and 390 days post-infection for the chronic infection. Statistically significant are indicated by horizontal bars ($p < 0.05$)

Supporting Table

Table S1: Serum pharmacokinetic parameters of different benznidazole treatments in dogs (n=8). Data are reported as median (interquartile range).

Parameter (unit)	Treatment 1	Treatment 2	Treatment 3
**Ka (h^{-1})**	-	3.44 (1.07 – 5.81)	0.88 (0.33 – 1.44)
**Cmax (µg/mL)**	8.11 (7.06 – 9.18)	6.39 (4.55 – 8.23)	5.29 (4.33 – 6.25)
**Tmax (h)**	-	1.62 (0.67 – 2.57)	3.77 (2.53 – 5.01)
**$t_{1/2a}$ (h)**	-	0.37 (0.05 – 0.68)	1.26 (0.59 – 1.93)
**$AUC_{0-\infty}$ (µg.h/mL)**	90.54 (64.22 – 116.87)	121.27 (83.81 – 158.73)	93.70 (73.15 – 114.25)
**F (%)**	-	100	100
**Vd (L)**	9.47 (8.04 – 10.89)	11.02 (7.83 – 14.22)	10.95 (8.27 – 13.64)
**CL (L/h)**	0.91 (0.68 – 1.13)	0.74 (0.43 – 1.06)	0.84 (0.68 – 1.01)
**Kel (h^{-1})**	0.09 (0.08 – 0.11)	0.07 (0.04 – 0.10)	0.08 (0.06 – 0.09)
**$t_{1/2el}$ (h)**	7.82 (5.48 – 10.16)	12.65 (7.36 – 17.93)	9.10 (7.06 – 11.13)

Note: Treatment 1, single intravenous dose 3.5 mg/Kg; Treatment 2, single intraperitoneal dose 3.5 mg/Kg; Treatment 3, single oral dose 3.5 mg/Kg; Ka, constant of absorption; C_{max}, maximum serum concentration; T_{max}, time to reach C_{max}; $t_{1/2a}$ absorption half-life; $AUC_{0-\infty}$, area under the serum concentration vs time curve from zero to infinity; F, bioavailability; Vd, volume of distribution; CL, total clearance; K_{el}, constant of elimination; $t_{1/2el}$, elimination half-life. *ANOVA test, $p < 0.05$.

References

1. European Medicines Agency (EMA). Guideline on Bioanalytical Method Validation (EMEA/CHMP/EWP/192217/2009). London, UK: EMA, 2011 https://www.ema.europa.eu/en/documents/scientific-guideline/guideline-bioanalytical-method-validation_en.pdf (Acessed, February 11, 2025).
2. Gabrielsson, J. and Weiner, D. (2012) Non-compartmental analysis. Methods Mol. Biol., 929, 377-389. DOI: 10.1007/978-1-62703-050-2_16.
3. R: A Language and Environment for Statistical Computing. Version 4.5.1. R Foundation for Statistical Computing; 2025. http://www.R-project.org/ (Acessed, July 10, 2024).
